# Mode of Progression in Smoldering Multiple Myeloma: A study of 406 patients

**DOI:** 10.21203/rs.3.rs-3378634/v1

**Published:** 2023-10-23

**Authors:** S Rajkumar, Nadine Abdallah, Arjun Lakshman, Shaji Kumar, Joselle Cook, Moritz Binder, Prashant Kapoor, Angela Dispenzieri, Morie Gertz, Martha Lacy, Suzanne HAYMAN, Francis Buadi, David Dingli, Yi Lin, Taxiarchis Kourelis, Rahma Warsame, P. Leif Bergsagel

**Affiliations:** Mayo Clinic; Mayo clinic; Mayo clinic; Mayo clinic; Mayo Clinic; Mayo Clinic; Mayo Clinic; Mayo Clinic; Mayo Clinic; Mayo Clinic; Mayo Clinic; Mayo Clinic; Mayo Clinic; Mayo Clinic; Mayo Clinic; Mayo Clinic; Mayo Clinic in Arizona

## Abstract

The approach to patients with high-risk smoldering multiple myeloma (SMM) varies among clinicians; while some advocate early intervention, others reserve treatment at progression to multiple myeloma (MM). We aimed to describe the myeloma-defining events (MDEs) and clinical presentations leading to MM diagnosis among SMM patients seen at our institution. We included 406 patients diagnosed with SMM between 2013–2022, seen at Mayo Clinic, Rochester, MN. The 2018 Mayo 20/2/20 criteria were used for risk stratification. Median follow-up was 3.9 years. Among high-risk patients who did not receive treatment in the SMM phase (n=71), 51 progressed by last follow-up; the MDEs included: bone lesions(37%), anemia(35%), hypercalcemia(8%), and renal failure(6%); 24% met MM criteria based on marrow plasmacytosis (≥60%) and/or free light chain ratio (>100); 45% had clinically significant MDEs (hypercalcemia, renal insufficiency, and/or bone lesions). MM diagnosis was made based on surveillance labs/imaging(45%), testing obtained due to provider suspicion for progression(14%), bone pain(20%), and hospitalization/ED presentations due to MM complications/symptoms(4%). The presentation was undocumented in 14%. A high proportion (45%) of patients with high-risk SMM on active surveillance develop end-organ damage at progression. About a quarter of patients who progress to MM are not diagnosed based on routine interval surveillance testing.

## Introduction

Smoldering multiple myeloma (SMM) is an asymptomatic clonal plasma cell disorder that represents an intermediate disease stage between monoclonal gammopathy of undetermined significance (MGUS) and multiple myeloma (MM). It is defined by serum monoclonal protein (IgG or IgA) ≥ 3 g/dL or urinary monoclonal protein ≥ 500 mg/24 hours and/or 10%-59% clonal plasma cells in the absence of end-organ damage attributable to the clonal plasma cell disorder,^1^ myeloma-defining biomarkers (serum involved to uninvolved free light chain ratio [FLCr] of ≥ 100 or > 1 focal lesion on magnetic resonance imaging [MRI]), or ALH amyloidosis.^2^ SMM accounts for about 14% of cases of MM with an estimated incidence of 0.9 cases per 100,000 persons in the US.^3^ The prevalence of SMM increases with advancing age, rising from about 0.5% in individuals ≥ 40 years to 1.6% in individuals ≥ 80 years.^4^ It is considered a heterogeneous disease entity which includes patients with variable risks of progression to active MM; in a subset of patients, the disease is biologically similar to MGUS and has a very low rate of progression, while in another subset, it is considered early MM and patients will progress to symptomatic MM within 2 years. Thus, risk stratification systems have been developed to distinguish between those 2 subsets.^5–7^ The Mayo 2018 20/2/20 system classifies patients into low-, intermediate-, or high-risk based on the presence of 0, 1, or ≥ 2 risk factors, respectively, of the following criteria: >20% bone marrow plasma cells (BMPCs), monoclonal (M) protein > 2 g/dL, and FLCr > 20.^6^ The 2020 International Myeloma Working Group (IMWG) risk stratification model further separates patients into 4 risk groups by incorporating cytogenetic abnormalities into the Mayo Clinic 2018 model.^7^ Historically, all patients with SMM were monitored until disease progression to symptomatic MM. However, the advent of highly efficacious novel therapeutic agents with less toxicity compared to the conventional cytotoxic chemotherapies, has led to the evaluation of early intervention strategies in patients with intermediate- and high-risk SMM. These include the low intensity and multiagent therapies to delay progression to symptomatic MM.^8–14^ Two randomized clinical trials have demonstrated the benefit of early intervention in delaying progression, with one study showing an overall survival (OS) advantage.^8, 9^ In 2013, the results of the phase 3 QuiRedex trial showed that early treatment with lenalidomide and dexamethasone was associated with a decrease in time to progression (TTP) and improvement in OS compared with observation alone in high-risk SMM patients;^9, 15^ benefits were sustained after 12.5 months of follow-up with (hazard ratios [HR]s) for TTP and OS of 0.28 (p < 0.001) and 0.57 (p = 0.03), respectively.^16^ Subsequently, the E3A06 ECOG-ACRIN phase 3 trial, comparing treatment with lenalidomide to observation in intermediate- or high-risk SMM patients, showed improvement in progression-free survival (PFS) (HR: 0.28, p = 0.002) with lenalidomide, but did not show an OS benefit.^8^ At this time, there is no consensus on the management of high-risk SMM outside of clinical trials, and practice varies even among clinicians within the same institution; while some advocate early intervention,^17^ others adopt a “watch and wait” approach, with the belief that close observation and serial testing can identify patients at imminent risk of progression, allowing initiation of treatment before serious end-organ damage occurs.^18, 19^ The aim of this study was to describe the myeloma-defining events (MDEs) and clinical presentations leading to the diagnosis of MM among SMM patients seen in our institution, including those who had early treatment and those who were observed and subsequently progressed to MM.

## Subjects and Methods

### Patient population:

This is a retrospective study including all patients diagnosed with SMM from August 2nd, 2013, until February 24th, 2022, who were seen at Mayo Clinic, Rochester, MN and have at least 6 months of follow-up data from SMM diagnosis. August 2013 was chosen as a cutoff for inclusion as this date corresponded to the initial publication on the results of the QuiRedex trial.^9^ Patients were identified from a preexisting database, and additional clinical, laboratory, and cytogenetic data were obtained by review of the electronic medical records. SMM was defined as ≥ 1 of the following: serum M protein ≥ 3 g/dL, urinary M protein ≥ 500 mg/24 hours, and/or ≥ 10% clonal BMPCs, without the “SliM CRAB” criteria: BMPCs ≥ 60%, involved/uninvolved FLCr ≥ 100 with involved FLC concentration > 100 mg/L, > 1 focal lesion involving the bone or bone marrow > 5 mm in size by magnetic resonance imaging (MRI), or any of the following attributable to the clonal plasma cell process: hypercalcemia (serum calcium > 11 mg/dL), renal insufficiency (serum creatinine > 2 mg/dL), anemia (hemoglobin < 10 g/dL or > 2 g/dL below the lower limit of normal), or ≥ 1 osteolytic bone lesions ≥ 5 mm in size on skeletal radiography, MRI, computed tomography (CT), or positron emission tomography/CT (PET/CT). Patients who met the criteria for systemic light chain amyloidosis were excluded. For all patients, we collected data on the management of SMM (observation versus treatment), the date of progression to symptomatic MM, the MDE at the time of progression, and the presentation that led to the diagnosis of MM. All included patients authorized the use of their medical record information for research purposes. The study was approved by the Mayo Clinic Institutional Review Board.

### Study outcomes:

The main aims of this study were to evaluate the 1) proportion of patients with SMM who are considered high-risk at the time of diagnosis, 2) proportion of patients with high-risk SMM who receive early treatment, 3) proportion of patients with high-risk SMM who progress to MM among those who receive early treatment and those who are managed with observation alone, and to describe the 4) MDEs at the time of progression and 5) clinical presentations that led to the diagnosis of active MM among SMM patients who progressed.

### SMM risk groups:

We used the 2018 Mayo 20/2/20 criteria to categorize patients into low-, intermediate-, and high-risk. Patients who had missing data for FLCr, %BMPCs, and/or M spike level were considered to have unknown risk. For analysis, we grouped patients into high-risk, non-high risk (low-risk and intermediate-risk), and unknown risk groups. Due to missing cytogenetic data for > 50% of patients, the 2020 IMWG risk stratification system was not used.

### MDE categories:

For patients who progressed to MM during their disease course, the MDE was categorized into the following non-mutually exclusive categories: 1) “bone lesions”, 2) “anemia”, 3) “hypercalcemia”, 4) “renal insufficiency”, 5) “MRI marrow lesions”, defined as > 1 focal lesion involving the bone marrow on MRI without other bone lesions on MRI, CT, PET/CT or skeletal radiography, and 6) “BMPC/FLCr criteria only”, defined as ≥ 60% clonal BMPCs and/or involved to uninvolved FLCr ≥ 100 (provided the involved FLC level was ≥ 100 mg/L) without end-organ damage or other MDEs.

### Presentation leading to MM diagnosis:

We reviewed the provider documentation and laboratory/imaging studies to determine the events leading to the diagnosis of active MM. We observed recurring themes which we classified into the following mutually exclusive categories: 1) “surveillance labs” if the diagnosis was made based on abnormalities on scheduled surveillance laboratory testing, satisfying MM diagnostic criteria (hypercalcemia, anemia, renal dysfunction, FLCr), 2) “surveillance imaging” if bone/bone marrow lesions satisfying MM criteria were detected on imaging obtained in the absence of symptoms, 3) “surveillance labs/imaging and symptoms” if in addition to (1) and/or (2), the patient has symptoms, for e.g. worsening fatigue, lightheadedness, and/or exertional dyspnea attributed to anemia, 4) “workup due to laboratory changes” if MM was diagnosed based on further workup (bone marrow biopsy or imaging) obtained due to changes observed on surveillance laboratory testing that were suspicious for progression (e.g. decrease in hemoglobin, increase in calcium/creatinine/monoclonal proteins otherwise not satisfying MDE criteria), 5) “workup for unrelated medical condition/symptom” if the diagnosis was made incidentally during workup of an unrelated medical condition or symptom, 6) “bone pain” if imaging obtained for evaluation of bone pain led to the diagnosis of MM, 7) “hospitalization/ ED due to MM complications/symptoms” if the patient presented to the emergency department (ED) or was hospitalized for evaluation or management of a symptom related to MM or end-organ damage (e.g. bone pain, symptoms of anemia, fracture, hypercalcemia, acute renal failure), and 8) “unknown”, if this data was unavailable.

### Statistical analysis:

Categorical data was expressed as frequency and percentages, and continuous data was summarized using median and interquartile range (IQR). The Kaplan-Meier method was used to estimate all time-to-event outcomes, including TTP, PFS, and OS; TTP was defined as the time from SMM diagnosis to the time of MM diagnosis. Patients who had not progressed were censored at their last follow up. PFS was defined as the time from SMM diagnosis until progression to MM or death from any cause (whichever occurred first). Patients who were alive and free of progression at their last follow-up were censored. OS was estimated from the time of diagnosis of SMM until death from any cause. Patients who were still alive were censored at their last follow-up. The frequency of SMM follow-up for each patient was defined as the difference (in months) between the dates of the last 2 SMM laboratory assessments. For patients who progressed to active MM, this was defined as the difference (in months) between the date of progression and the date of the last SMM laboratory assessment prior to progression. All data analysis was performed using the jmp statistical software (SAS, Cary, NC).

## Results

### Patient population and outcomes in the overall cohort:

We included 406 patients diagnosed with SMM between August 2nd, 2013, and February 24th, 2022. The baseline characteristics are shown in Table 1. The median age was 66 (range: 31–89) years, and 58% were male. Ninety patients (22%) were high-risk, 116 (29%) were intermediate-risk, and 110 (27%) were low-risk; risk was unknown in 90 (22%) patients. The median follow-up was 4.4 (95%CI: 3.4–4.8), 3.4 (95%CI: 3.0–3.9), and 5.0 (95%CI: 4.2–6.1) years in patients who were high-risk, non high-risk, and unknown risk, respectively. At the time of analysis, 159 patients (39%) had progressed to MM by last follow-up. The median TTP was 2.6 (95%CI: 1.8–3.6) years in the high-risk group, 7.0 (95%CI: 5.9–7.9) years in non high-risk group, and 4.5 (95%CI: 3.1–7.2) years in patients with unknown risk. Forty patients (10%) had died by last follow-up. The median PFS was 2.6 (95%CI: 1.7–3.2), 6.7 (95%CI: 5.5–7.9), and 4.3 (95%CI: 3.0–6.5) years in the 3 groups, respectively. The median OS was not reached. The frequency of follow-up for all SMM patients ranged between 1 and 18 months; the follow-up interval could not be determined for 50 patients who had follow-up outside of Mayo Clinic and had insufficient data in our medical record system to estimate this interval. The median follow-up frequency was 3 (IQR: 2–4) months for high-risk SMM patients, and 3 (IQR: 3–6) months for non high-risk and for unknown risk SMM patients.

### MDEs and presentations in high-risk SMM:

**Figure 1:**
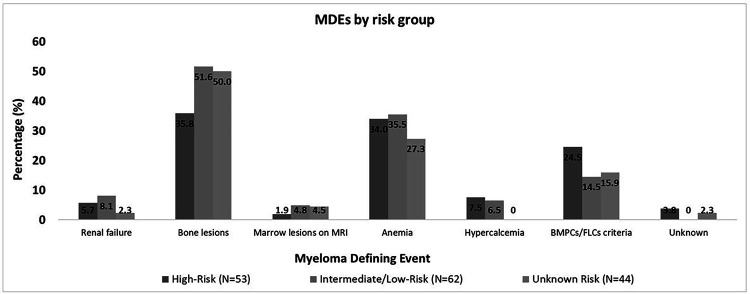
MDEs by SMM risk group. MDEs in patients with high-risk, intermediate- or low-risk, and unknown risk SMM. *Abbreviations: BMPCs: bone marrow plasma cells, FLCs: free light chains, MDE: myeloma-defining event, MRI: Magnetic Reasonance Imaging, SMM: smoldering multiple myeloma.*

Among patients with high-risk SMM (90 patients), 71 (79%) were observed and 19 (21%) received anti-myeloma treatment at the SMM stage (Table 2) after a median of 1.4 (range: 0–32.0) months from diagnosis. Among those who received treatment (19 patients), 2 (11%) had progressed to MM by last follow-up; both patients had received treatment within 1 month from SMM diagnosis. One patient progressed after 0.9 years from diagnosis; the MDE and presentation leading to diagnosis are not documented. The other patient progressed after 5.2 years from diagnosis with ≥ 60% BMPCs in the absence of end-organ damage. In this patient, bone marrow biopsy confirming progression to MM was obtained due to increasing paraproteins on follow-up laboratory testing. Among patients who were observed, 51 (72%) had progressed by their last follow-up with a median TTP of 2.2 (95%CI: 1.3–2.6) years. The MDEs were bone lesions in 19 patients (37%) patients, anemia in 18 patients (35%), hypercalcemia in 4 patients (8%), and renal failure in 3 patients (6%); 12 patients (24%) had BMPCs/FLCr criteria only. The MDE was unknown in 1 patient. Twenty-three patients (45%) had clinically significant MDEs (hypercalcemia, renal insufficiency, any/or bone lesions), and 17 patients (33%) patients had > 1 MDE. Among the three patients with renal insufficiency, 1 progressed to end-stage renal disease, while in the other 2 patients, the renal function returned to baseline. Three patients (6%) had compression fractures and another 2 patients (4%) had other rib fractures. The MDEs in different risk groups are shown in Fig. 1.

**Figure 2:**
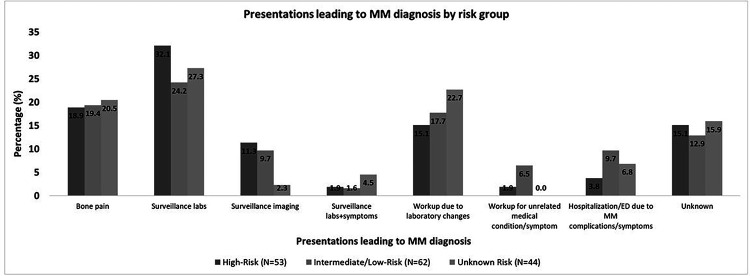
Presentations at progression by SMM risk group. Presentations leading to the diagnosis of MM in patients with high-risk, intermediate- or low-risk, and unknown risk SMM. *Abbreviations: ED: emergency department, MM: multiple myeloma, SMM: smoldering multiple myeloma.*

Among the patients on active surveillance who progressed by last follow-up (51 patients), the presentations leading to diagnosis of MM were: surveillance labs/surveillance imaging in 23 patients (44%), workup due to laboratory changes in 7 patients (14%), bone pain in 10 patients (19%), workup for an unrelated symptom in 1 patient, and hospitalization/ED due MM complications/symptoms in 2 patients (4%); 1 patient was hospitalized for symptomatic hypercalcemia (altered mental status), and the other patient was hospitalized with hypercalcemia and acute renal failure. In the latter patient, renal function returned to baseline. The presentations leading to the diagnosis of MM was undocumented in 7 patients (14%), The presentations leading to MM diagnosis in the different risk groups are shown in Fig. 2.

### MDEs and presentations in non-high-risk patients:

Among patients with non-high risk SMM (226 patients), 35 (15%) received treatment for SMM after a median of 4.0 (range: 0–51.1) months from diagnosis, and 191(85%) were observed (Table 2). Among those 35 patients who received treatment, 3 patients (9%) progressed by their last follow-up after 4.8, 6.0, and 7.6 years from diagnosis. The MDEs in these 3 patients were bone lesions, anemia and bone lesions, and BMPCs/FLCr criteria only; two were diagnosed with MM based on surveillance labs/surveillance imaging, and another patient was diagnosed based on workup due to laboratory changes. Among those who were observed (191 patients), 59 (31%) had progressed by their last follow-up with a median TTP of 6.7 (95%CI: 4.9–7.9) years. The MDEs were bone lesions in 30 patients (51%), anemia in 21 patients (36%), hypercalcemia in 4 patients (7%), renal insufficiency in 5 patients (8%), and BMPCs/FLCr criteria only in 8 patients (14%). Twenty (34%) had > 1 MDE, and 35 (59%) had clinically significant MDEs (Fig. 1). Four patients (7%) had compression fractures. Among those who had renal insufficiency (5 patients), renal function returned to baseline in 1 patient only.

The presentations leading to MM diagnosis were surveillance labs/surveillance imaging in 19 patients (32%), workup due to laboratory changes in 10 patients (17%), bone pain in 12 patients (20%), workup for unrelated medical condition/symptom in 4 patients (7%),, unknown presentation in 8 patients (14%), and hospitalization/ED due MM complications/symptoms in 6 patients (10%); 1 patient presented to the ED with bone pain, and 5 patients were hospitalized for: bone pain and compression fracture (1), renal failure (1), hypercalcemia and renal failure (1), hypercalcemia with renal failure and severe anemia (1), and symptomatic anemia (1); among those, 2 patients died after 2 and 4 months from MM diagnosis.

### MDEs and presentations in unknown-risk SMM:

In 90 patients, the risk was unknown. Among those, 7 patients (8%) received treatment for SMM and 83 patients (92%) were observed (Table 2). Among those who received treatment, 2 progressed by their last follow-up after 2.0 and 3.7 years from diagnosis. Among those who were observed, 42 patients (51%) progressed by last follow-up with a median TTP of 4.3 (95%CI: 2.9–7.3) years from diagnosis. The MDEs and presentations for these patients are shown in Figs. 1 and 2, respectively.

## Discussion

Patients with high-risk SMM constitute about a third of SMM patients at diagnosis.^6^ Since 2013, 2 phase 3 clinical trials have shown that early intervention in high-risk SMM delays progression to MM, including 1 study which demonstrated a survival benefit.^8, 15^ Over a 10-year period since the initial results of the QuiRedex study were reported,^9^ 90 patients with high-risk SMM were seen in our institution, 21% of whom received early intervention, while the rest were observed. This reflects the variability in management of this subset in the real-world setting. During the study period, 72% of patients who were observed had progressed to MM; among patients who received early treatment, 11% progressed to MM. About half of patients who progressed had clinically significant end-organ damage including 1 patient who progressed to end-stage renal disease. Similar to the findings in the ECOG-ACRIN SMM trial,^8^ bone lesions were the most common MDE, found in over a third of patients. The frequency of SMM follow-up in our cohort was variable, ranging between 1 and 18 months, which reflects real-world practice. Both high- and non high-risk patients had a median follow-up frequency of 3 months. Among patients with high-risk SMM, testing obtained during follow up led to the diagnosis of MM in about 60% of cases, while approximately 20% were diagnosed after presenting with bone pain, and 2 patients were hospitalized with hypercalcemia and acute renal failure. The non high-risk patients who were observed had a lower rate of progression and a longer TTP to MM compared to the high-risk patients. However, more than half of those who progressed had clinically significant end-organ damage, and all but one patient who presented with renal failure developed chronic renal disease. Similar to the findings in the high-risk group, bone pain accounted for 20% of presentations leading to MM diagnosis, and 5 patients were hospitalized due to MM complications including 2 patients who died shortly after diagnosis.

Our results, based on real-world data, demonstrate that the majority of patients with high-risk SMM will eventually progress to MM as predicted by their baseline risk assessment, and that the end-organ damage may not be preventable even when patients are followed up in tertiary care centers. A subset of patients will have bone lesions and/or renal failure, which may not be reversible in all. In addition, in about a quarter of patients, labs and/or imaging obtained at follow-up are not sufficient to detect progression, Although interpretation is limited by small sample size, patients who received early intervention appeared to have a lower rate of progression to MM during the study period compared to those who were observed. The data supporting early intervention in high-risk patients comes from two clinical trials which used different criteria for risk stratification; the QuiRedex study^9^ used immunophenotype-based risk stratification which is not currently adopted in clinical practice. In addition, it is likely that a subset of patients enrolled on the QuiRedex study would be reclassified as MM with the use of sensitive skeletal imaging. The ECOG-ACRIN study, which used more sensitive imaging to diagnose SMM did not show a survival benefit, but follow-up was only 35 months.^8^ Nevertheless, the morbidity associated with progression to MM seen in our study, the benefit in delaying progression reported in the 2 trials, and the lack of evidence for emergence of resistant clones, all justify early intervention in patients with high-risk SMM. Until the randomized data for triplet and quadruplet regimens emerges, lenalidomide-based therapy or observation alone are being recommended outside of clinical trials. In this study, we observed that some patients who were non-high risk at diagnosis also developed clinically significant end-organ damage, including long-term consequences. It is possible that a fraction of those patients would have met the criteria for high-risk disease during follow up and would have also benefitted from early intervention.

This study has several limitations: due to the retrospective nature of the study, some patients had missing data for risk stratification, MDE, and presentation at the time of progression to MM. Less than 50% of patients had cytogenetic data, so we could not use the IMWG criteria for risk stratification.^20^ In this study, we had available data to determine the frequency of SMM follow-up for 88% of patients, but we did not report the imaging type and frequency, as data was missing for a large number of patients.

Among patients who received treatment, there was variability in the timing, type, intensity (single agent vs doublet vs triplet), and duration of treatment. The median time to treatment initiation was 1.4 and 4.0 months in the high- and non-high-risk groups, respectively. However, some patients started treatment many months, and even several years, after the diagnosis of SMM. This is largely attributed to changes in laboratory parameters causing migration to a higher risk group during follow-up, and to the timing of trial availability and/or trial access.

Despite limitations, our study provides important information on the presentations and MDEs in patients with SMM followed in a real-world setting who subsequently progress to MM. We recommend shared decision making and discussion of pros and cons regardless of whether early intervention is used or not. We believe this manuscript provides additional data needed to assist patients and physicians in this regard, and complements data from randomized trials.

## Conclusion

At this time, there is variability in the management of patients with high-risk SMM in the clinical setting. Despite clinical trial data suggesting benefit from early intervention, the majority of patients with SMM are observed. Our experience over a 10-year period suggests that clinically significant end-organ damage may not be preventable with expectant monitoring alone, and that progression to MM is not always detected by follow-up testing. These findings may provide support for early intervention in high-risk patients. Efforts to further refine the current risk stratification systems will better delineate the subset of patients who would benefit from early intervention in the future.

## Figures and Tables

**Table 1:** Baseline characteristics

Baseline characteristics (N=406)	Median (interquartile range)	N (%)
**Age**	66 (57–72)	
**Sex (male)**		236 (58)
**Race/ethnicity**		
African/African American		14 (3)
Asian		13 (3)
Hispanic		6 (1)
Native American		2 (<1)
Non-Hispanic white		343 (84)
White-unknown		5 (1)
Other		4 (1)
Unknown		19 (5)
**Hemoglobin (g/dL)**	13 (11.8–14)	
**Calcium (mg/dL)**	9.3 (9–9.7)	
**Creatinine (mg/dL)**	0.9 (0.8–1.2)	
**M spike (g/dL)**	1.7 (1.0–2.4)	
**Involved light chain**		
K		266 (66)
L		140 (34)
**Kappa free light chain (mg/dL)**	14.3 (4.6–33.6)	
**Lambda free light chain (mg/dL)**	10.4 (3.6–37.4)	
**Bone marrow plasma cell (%)**	20 (14–25)	
**Fluorescence in situ hybridization**		
t(11; 14)		
Present		41 (10)
Absent		105 (26)
Missing		260 (64)
t(4;14)		
Present		25 (6)
Absent		69 (17)
Missing		312 (77)
t(14;16)		
Present		9 (2)
Absent		70 (17)
Missing		327 (81)
t(14;20)		
Present		6 (1)
Absent		54 (13)
Missing		346 (85)
Trisomy		
Present		99 (24)
Absent		193 (48)
Missing		114 (28)
Deletion13q/Monosomy 13		
Present		97 (24)
Absent		82 (20)
Missing		227 (56)
Deletion17p/Monosomy 17		
Present		14 (3)
Absent		154 (38)
Missing		238 (59)
1p deletion		
Present		12 (3)
Absent		93 (23)
Missing		301 (74)
1q gain		
Present		74 (18)
Absent		96 (24)
Missing		236 (58)
c-myc abnormality		
Present		13 (3)
Absent		68 (17)
Missing		325 (80)

**Table 2.** Treatments by SMM risk group

Treatment	Low-Risk (N=110)	Intermediate-Risk (N=116)	High-Risk (N=90)	Unknown Risk (N=90)
**Observation**	90	101	71	83
**Standard treatment**	10	8	9	5
CYBORD	2	0	1	0
DKRD	0	0	1	0
DRD	0	1	2	0
VRD	3	0	1	1
IRD	1	0	0	0
RD	4	4	3	1
KD	0	0	0	1
R	0	3	1	1
V	0	0	0	1
**Clinical trial**	10	7	10	2
DKRD	0	2	4	0
DRD	0	1	1	0
RD+Anakinra	4	1	2	0
R+neoadjuvant vaccine	2	0	0	0
RD	2	2	2	1
R	2		1	1
D	0	1	0	0

Abbreviations: CYBORD: cyclophosphamide, bortezomib, dexamethasone, D: daratumumab, DKRD: daratumumab, carfilzomib, lenalidomide, dexamethasone, DRD: daratumumab, lenalidomide, dexamethasone, IRD: ixazomib, lenalidomide, dexamethasone, KD: carfilzomib, dexamethasone, R: lenalidomide, RD: lenalidomide, dexamethasone, V: bortezomib, VRD: bortezomib, lenalidomide, dexamethasone.

## Data Availability

The data generated in this study is available upon request from the corresponding author.
